# Transcriptomics and Metabolomics Signatures of Fat Deposition Following Orchiectomy in Yak

**DOI:** 10.3390/ani16121825

**Published:** 2026-06-12

**Authors:** Lin Xiong, Jie Pei, Qianyun Ge, Zhiqiang Ding, Yandong Kang, Chao Chen, Ruichao Wei, Xian Guo

**Affiliations:** 1 Animal Science Department, Lanzhou Institute of Husbandry and Pharmaceutical Sciences, Chinese Academy of Agricultural Sciences, Lanzhou 730050, China; xionglin@caas.cn (L.X.); peijie@caas.cn (J.P.); geqianyun@caas.cn (Q.G.); dingziqiang1997@163.com (Z.D.); kangyandong0901@163.com (Y.K.); chenchaocaas@163.com (C.C.); ruichaowei2001@163.com (R.W.); 2 Key Laboratory of Yak Breeding in Gansu Province, Lanzhou 730050, China; 3 Key Laboratory of Animal Genetics and Breeding on Tibetan Plateau, Ministry of Agriculture and Rural Affairs, Lanzhou 730050, China

**Keywords:** yak, castration, gene regulation, fat deposition, fatty acid, multi-omics

## Abstract

Fat deposition plays an important role in yak metabolism, reproduction, and meat quality, and male yaks are often castrated to facilitate management and improve production performance. The effect of castration on the characteristics of fat deposition in male yaks and the molecular mechanisms of action was explored in this study. The subcutaneous fat thickness in castrated and common male yaks was measured, further the content of fatty acids in yak subcutaneous fat was detected using gas chromatography-mass spectrometer (GC-MS); the transcriptome, metabolome in the yak subcutaneous fat were detected using mRNA-Sequencing, ultra-high performance liquid chromatography-tandem mass spectrometry (UHPLC-MS/MS), respectively; the integrative analyses of differentially expressed genes (DEGs), different metabolites (DMs), fatty acids and fat thickness were carried out. The results showed that castration can strengthen the ability of fat deposition and improve the content of fatty acids, especially PUFAs, in male yaks, and both transcriptome and metabolome were significantly different between castrated male yaks and common male yaks. The effect of castration on the male yak fat deposition was closely related to the PPAR signaling pathway, citrate cycle, and insulin resistance. Data suggests that *FASN*, *ACACA*, *AGPAT2*, *ACLY*, *ACSL5*, *SCD*, *GSK3B*, and *SLC2A4* may be the crucial control genes for the fat amount in yaks, and that *FADS2*, *LPL*, and *ACSL4* may be the crucial control genes for the polyunsaturated fatty acids (PUFAs) content in yak adipose tissue. Further functional studies will be conducted to determine the specific role of each gene in regulating fat deposition and fatty acid composition in yaks.

## Simple Summary

Castration, known to influence fat deposition and meat quality, is the common practice in cattle husbandry, and the effect of castration on male yak fat deposition was explored in this study. The finding showed castrated yaks had thicker subcutaneous fat and higher levels of beneficial fatty acids, particularly polyunsaturated fatty acids. Further, the key genes that controlled the fat amount and the content of polyunsaturated fatty acids in yaks were identified, and several crucial biological pathways linked to the effect of castration on yak fat deposition were screened out. These findings can help farmers raise yaks more effectively, improve meat quality for consumers, and support healthier breeding practices. Ultimately, both yak farming and the people who rely on yak products will benefit from this research.

## 1. Introduction

Beef cattle possess a fast growth rate and a high percentage of lean meat, but they are difficult to manage due to their fierce temperament and frequent estrus. In traditional production systems, orchidectomy (also known as castration), which involves the surgical removal of the testes, is commonly used [1,2]. Because castrated animals are unable to produce gametes, castration prevents the reproduction of undesirable or genetically inferior individuals. It also reduces aggressiveness and makes cattle easier to feed and manage [3,4]. Furthermore, castration helps improve beef yield and quality and can enhance meat grade [5]. In bulls, castration redirects the nutrients and energy from feed away from the reproductive system, concentrating them in muscle and thereby increasing meat production. During the fattening period, castrated cattle accumulate more intramuscular fat, which enhances meat tenderness [6,7]. From the perspective of molecular biology, the regulatory pathways of certain growth traits in castrated cattle differ from those of common male cattle [8,9]. Therefore, reasonable utilization of the differences between castrated cattle and bulls, understanding the mechanisms for these differences, and fully utilizing excellent genetic resources will provide an effective strategy to improve cattle production efficiency and accelerate the process of beef cattle breeding.

Yak (*Bos grunniens*), with approximately 20 million in the world, is one of the unique cattle mainly inhabiting the Tibetan Plateau [10]. Because of the harsh natural conditions and the scarcity of resources, yak plays an essential role in the daily life of local residents by providing animal-derived food, transport, shelter, and fuel [11,12]. Triglycerides, as an important molecule for energy storage, are the main fat composition in yak, and are vital for their life activities [13]. Moreover, the type and amount of fat are the key factors that influence the meat quality in yak [14], such as appearance, tenderness, texture, and flavor. Therefore, fat deposition is currently a key research focus in yak production. Numerous factors, including genetics, diet, sex, and breed, have been shown to influence this trait in yak [15,16]. As semi-wild domestic animals, male yaks are typically castrated unless kept for breeding. Castration reduces aggressive and combative behaviors, resulting in a more docile temperament. However, the effects of castration on fat deposition in yak remain poorly understood. We hypothesize that castration triggers molecular and genetic mechanisms that regulate the amount and composition of subcutaneous fat in yaks. Investigating the characteristics and regulatory mechanisms of fat deposition in castrated male yaks could provide a theoretical foundation for the deep processing of yak meat and the molecular breeding of yaks.

In the present study, a relatively comprehensive study was carried out to characterize the fat deposition in castrated male yaks and explore the molecular mechanism underlying the effect of castration on fat deposition in yaks. First, the thickness of subcutaneous fat in the waist and back of castrated male yaks and common male yaks was measured, and the fatty acid content in yak subcutaneous fat was detected by gas chromatography-mass spectrometer (GC-MS). Next, transcriptome and metabolome in the subcutaneous fat of castrated male yaks and common male yaks were detected using mRNA-Sequencing (mRNA-Seq) and ultra-high performance liquid chromatography-tandem mass spectrometry (UHPLC-MS/MS), respectively. Differentially expressed genes (DEGs) and different metabolites (DMs) in the yak subcutaneous fat were identified; further, the function of DEGs and DMs was annotated by gene ontology (GO) and Kyoto encyclopedia of genes and genomes (KEGG) analysis, respectively. Finally, the pivotal regulatory genes and signaling pathways driving the effect of castration on fat deposition in male yaks were explored based on the correlation analysis among fat phenotype data, DEGs, and DMs. These results could elucidate the molecular mechanisms regulating fat deposition and provide a theoretical basis for breeding programs aimed at improving yak meat quality.

## 2. Materials and Methods

### 2.1. Animals and Samples Collection

Twelve male, four-year-old Tianzhu white yaks were used as the experimental animals. Of them, six yaks were castrated by orchidectomy at two years old. These yaks fasted for 12 h before castration. The scrotum was then cleaned, shaved, and disinfected. The operator’s hands and surgical instruments were also strictly sterilized. After the scrotum was incised, the testis was extruded through the incision. The spermatic cord was isolated and held, then twisted from slow to fast in a consistent direction until the cord, along with blood vessels and nerves, was ruptured, which facilitated the removal of the testis. Postoperatively, the wound was kept clean. Penicillin powder was applied inside the wound, and the wound surface was painted with iodine tincture. All yaks were fed under natural grazing conditions and can freely eat grass and drink water on the natural pasture of Tianzhu County in Gansu Province. These castrated male yaks were the experimental group (EG and the common male yaks were the control group (CG). All animal procedures were conducted in strict accordance with animal welfare regulations and were approved by the Ethics Committee of Lanzhou Institute of Husbandry and Pharmaceutical Sciences, Chinese Academy of Agricultural Sciences (Permit No. 2024-44). All yaks were slaughtered through electrical stunning at four years old. Approximately 20 g of subcutaneous fat was collected from the surface of the longissimus dorsi at the 12th~13th rib level of each yak. The sample was then divided into ten equal aliquots, placed into 2 mL cryovials, and immediately frozen in liquid nitrogen for subsequent analyses.

### 2.2. Measurement of Subcutaneous Fat Thickness

Subcutaneous fat thickness was measured using a vernier caliper (Hengliang Inc., Shanghai, China) within 10 min post-slaughter. Because there are differences in the thickness of subcutaneous fat in yaks’ different parts, the measurement site must be representative in order to achieve uniformity and accuracy. In this study, the subcutaneous fat on the back (both sides of the dorsal midline at the 5~6 thoracic vertebrae) and on the waist (both sides of the midline at the cruciate region) of yaks were selected for measurement. After the half carcass of yaks was cut, the thickness of subcutaneous fat on the specified area was measured in a short time.

### 2.3. RNA Extraction, Library Preparation, and Sequencing

The total RNA in fat samples was extracted using the MJZol total RNA extraction kit (Meiji Biomedical Technology Co., Ltd., Shanghai, China) according to the manufacturer’s instructions. RNA purity and concentration were quantified using a NanoDrop2000 ultramicro spectrophotometer (Thermo Fisher Scientific, Santa Clara, CA, USA). Agarose gel electrophoresis was used to assess RNA integrity, and the 5300 bioanalyzer (Agilent Technologies, Santa Clara, CA, USA) was used to determine RQN value. High-quality RNA (OD260/280 = 1.8~2.2, OD260/230 ≥ 2.0, RQN ≥ 6.5, 28S:18S ≥ 1.0, >1 μg) was selected to construct a sequencing library, and mRNA purification, reverse transcription, and library construction were performed using the Illumina^®^ Stranded mRNA Prep, Ligation (Illumina, San Diego, CA, USA). Magnetic beads with Oligo (dT) were used to isolate mRNA from total RNA. The mRNA was randomly fragmented into small fragments of approximately 300 bp using the fragmentation buffer. Using reverse transcriptase, the first-strand cDNA was synthesized with mRNA as template, followed by the second-strand synthesis. End Repair Mix was added to convert the double-stranded cDNA into blunt ends, followed by the addition of an A base to the 3′ ends. The adapter-ligated products were purified and size-selected, and the size-selected products were subjected to PCR amplification. Finally, the library was obtained after the purification and was sequenced on the NovaSeq X Plus platform (Illumina, San Diego, CA, USA).

Raw read was trimmed and controlled using fastp software (Version 0.23.4), then the obtained clean read was aligned to the yak reference genome (LU_Bosgru_v3.0, https://mart.ensembl.org/Bos_grunniens/Info/Index, accessed on 25 June 2025) using HISAT2 (Version 2.2.1). The mapped reads for each sample were assembled using StringTie (Version 2.2.1). Transcript expression level was calculated according to fragments per kilobase of exon model per million mapped fragments (FPKM) method. Gene abundance was quantified using RSEM software (Version 1.3.3). Differential expression analysis was performed using DESeq2 (Version 1.42.0). A fold change (FC) >2 or <0.5, and an adjusted *p*-value (*p*-adjust) < 0.05 were set as the threshold for defining DEGs. GO enrichment analyses for DEGs were performed by comparing the DEGs against the GO database (http://www.geneontology.org/) using Goatools (Version 1.4.4), and KEGG enrichment analyses for DEGs were performed by comparing the DEGs against the KEGG database (http://www.genome.jp/kegg/, accessed on 8 June 2026) using Python scipy. A *p*-adjust value < 0.05 was considered to indicate statistically significant enrichment.

### 2.4. Metabolite Extraction and Mass Spectrum (MS) Data Collection

Fifty mg of the fat sample was put into a 2 mL centrifuge tube, and a 6 mm grinding bead was put into the tube too. Four hundred μL of the mixed solution (methanol:water, 4:1, *v*:*v*) containing 0.02 mg/mL L-2-chlorophenylalanine as the internal standard was used for metabolite extraction. The sample was ground at −10 °C for 6 min using a Wonbio-96c frozen grinder (Shanghai Wanbo biotechnology Co., Ltd., Shanghai, China), followed by ultrasonic extraction in an SBL-10DT ultrasonic cleaning machine (Xinzi Biotechnology Co., Ltd., Ningbo, China) at 5 °C for 30 min. Then the mixture was left for 30 min at −20 °C, followed by being centrifuged at 13,000 r/min for 15 min using 5430R high-speed refrigerated centrifuge (Eppendorf, Hamburg, Germany). Finally, the supernatant was transferred out and filtered into a vial for LC-MS/MS analysis. Quality control (QC) samples were prepared by pooling equal volumes of extraction from all samples and were subsequently subjected to the same processing and analytical procedure as the test samples.

A Triple TOF 6600 mass spectrometer (SCIEX, Framingham, MA, USA) coupled to an ExionLC AD liquid chromatography system (AB SCIEX, Framingham, MA, USA) was used to collect metabolites data, and the chromatographic separation of prepared samples was performed on an ACQUITY HSS T3 column (100 mm × 2.1 mm × 1.8 μm; Waters, Milford, MA, USA). The mobile phases were consisted of A water containing 0.1% formic acid and acetonitrile (95.0:5.0, *v*:*v*) and B acetonitrile contained 0.1% formic acid, isopropanol and water (47.5:47.5:5.0, *v*:*v*:*v*). The separation gradient on positive ion mode was as follows: 0.0~20.0% B from 0.0 to 3.0 min, 20.0~35.0% B from 3.0 to 4.5 min, 35.0~100.0% B from 4.5 to 5.0 min, 100.0% B from 5.0 to 6.3 min, 100.0~0.0% B from 6.3 to 6.4 min, 0.0% B from 6.4 to 8.0 min. The separation gradient on negative ion mode was as follows: 0.0~5.0% B from 0.0 to 1.5 min, 5.0~10.0% B from 1.5 to 2.0 min, 10.0~30.0% B from 2.0 to 4.5 min, 30.0~100.0% B from 4.5 to 5.0 min, 100.0% B from 5.0 to 6.3 min, 100.0~0.0 B% from 6.3 to 6.4 min, 0.0% B from 6.4 to 8.0 min. The flow rate and column temperature were 0.40 mL/min and 40 °C, respectively. MS parameter was set as follows: source temperature 500 °C, curtain gas 35 psi, ion source gas1 50 psi and gas2 13 psi, ion-spray voltage floating −4500 V in negative mode and 5500 V in positive mode, declustering potential 80 V, collision energy 20~40~60 eV. Data-dependent acquisition (DDA) mode was employed, and the mass range was 50~1200 m/z.

Raw data acquired by LC-MS/MS were imported into Progenesis QI software (version 3.1) for peak picking, alignment, and normalization. Metabolites were identified by comparing the obtained MS data against the HMDB (http://www.hmdb.ca/) and Metlin (https://metlin.scripps.edu/) databases. Principal component analysis (PCA) and orthogonal least partial squares discriminant analysis (OPLS-DA) were performed using the R package ropls (Version 1.6.2), and model stability was evaluated using 7-cycle interactive validation. Variable importance in projection (VIP) values derived from OPLS-DA and Student’s *t*-test were used to screen DMs. Metabolites with VIP > 1, *p* < 0.05 were considered as DMs. The identified DMs were then subjected to KEGG enrichment analysis using a Python package, and pathways with a corrected *p*-value < 0.05 were considered significant enrichment.

### 2.5. Fatty Acids Determination

A 100 mg fat sample and a small steel ball were placed into a 2 mL grinding tube, followed by the addition of 1 mL mixed solution (dichloromethane:methanol, *v*:*v*, 1:1). The tube was ground at 50 Hz for 3 min using a Wonbio-96c cryogenic grinding machine (Wanbai biotechnology Co., Ltd., Shanghai, China) and then subjected to ultrasonic extraction for 15 min at low temperature using an SBL-10DT ultrasonic cleaning machine (Xinzhi Biotechnology Co., Ltd., Ningbo, China). The mixture was stood for 15 min at −20 °C, followed by centrifuging for 10 min at 13,000 r/min, 4 °C. A 500 μL supernatant was transferred to a 1.5 mL EP tube and was dried under nitrogen using a JXDC-20 nitrogen blower (Jinsin industrial development Co., Ltd., Shanghai, China). Boron fluoride-methanol solution was added to derive the fatty acids. The solution was stirred for 30 s and incubated at 60 °C for 30 min, then the fatty acids were translated into fatty acid methyl esters (FAMEs). After cooling, 0.5 mL *n*-hexane was added, and the solution was stirred for 30 s and centrifuged at 13,000 r/min for 10 min. Finally, 100 μL of the upper layer solution was transferred to a vial for analysis.

FAMEs were analyzed using an Agilent 8890B-7000D GC-MS (Agilent, Santa Clara, CA, USA) coupled with an Agilent DB-FastFAME capillary column (20 m × 0.18 mm × 0.20 µm) (Agilent, CA, USA). The carrier gas was helium at 1 mL/min. Column temperature program was as follows: hold at 80 °C for 30 s, increased to 175 °C at 70 °C/min, then to 230 °C at 8 °C/min, and hold at 230 °C for 2 min. The injection volume was 1 µL, and the inlet temperature was set at 230 °C. The mass spectrometer was operated under the following conditions: the ion source temperature was 230 °C; the quadrupole temperature was 150 °C; the scanning mode was selected. FAMEs were identified and quantified using Masshunter software (Version 10.0.707.0). Quality control (QC) samples prepared by pooling aliquots of all test samples were used to evaluate the stability of the analytical system. The concentration of 36 FAMEs was calculated by an external standard method. The analytes were determined based on their retention times, and the absolute content of fatty acids was calculated by corresponding FAMEs.

### 2.6. Real-Time Quantitative PCR (RT-qPCR)

A total of 6 samples from the EG and CG were selected for *RT-qPCR* analysis. Four DEGs (*SCD*, *FASN*, *AGPAT2*, and *LIPE*) from different pathways were randomly selected to confirm mRNA-Seq results. The information on primers for the 4 selected DEGs in yak subcutaneous fat of the two groups is shown in Table S1. Total RNA in fat samples was extracted, and the concentration and the rate of OD260/OD280 were measured using a NanoDrop 2000 spectrophotometer (Thermo Scientific, Waltham, MA, USA). The RNA integrity was assessed by agarose gel electrophoresis. Subsequently, reverse transcription was performed in a GeneAmp^®^ PCR System 9700 (Applied Biosystems, Waltham, MA, USA) using the TransScript All-in-One First-Strand cDNA Synthesis SuperMix for qPCR kit (TransGen Biotech Co., Ltd., Beijing, China). Reaction mixture contained 0.5 µg total RNA, 2 µL 5× TransScript All-in-one SuperMix for qPCR, and 0.5 µL gDNA Remover, and the mixture volume was adjusted to 10 µL using nuclease-free water. Reverse transcription reaction was carried out according to the following conditions: 42 °C for 15 min, followed by 85 °C for 5 s. After the reaction, 90 µL nuclease-free water was added to the cDNA product, and the sample was stored at −20 °C. The qPCR was performed using the PerfectStart™ Green qPCR SuperMix kit on a LightCycler^®^ 480 II real-time PCR system (Roche, Basel, Basel-Stadt, Switzerland). Reaction mixture consisted of 5 µL 2 × PerfectStart™ Green qPCR SuperMix, 0.2 µL 10 µmol/L forward primers, 0.2 µL 10 µmol/L reverse primers, 1 µL cDNA, and 3.6 µL nuclease-free water. The thermal cycling protocol was as follows: initial denaturation at 94 °C for 30 s, followed by 45 cycles at 94 °C for 5 s and 60 °C for 30 s. After cycling, the specificity of amplified products was assessed by melting curve analysis. The fluorescence signal was collected when the temperature gradually increased from 60 °C to 97 °C. The *β-actin* gene was used as the reference gene in yak subcutaneous fat, and the relative expression level of selected genes was determined by the 2^−∆∆Ct^ method.

### 2.7. Statistical Analysis

Fatty acid content was analyzed using an independent samples Student’s *t*-test in SPSS Statistics 22 (IBM, Armonk, NY, USA). The correlation between subcutaneous fat thickness, fatty acids content, DMs abundance, and DEGs expression level was carried out by Pearson correlation analysis using SPSS 22 software, too. A *p*-value < 0.05 was considered statistically significant, and a correlation coefficient > 0.8 was defined as a high correlation. The integrative analysis for transcriptome and metabolome was carried out using Python (Version 1.0.0). Correlation network analysis was conducted by calculating correlation coefficients between DMs and DEGs, and then network diagrams were generated to display the interaction relationships among these molecules. The mutual KEGG pathways for these DEG and DM enrichments were identified using the hypergeometric distribution algorithm. Benjamini–Hochberg (BH) method was applied to adjust *p*-values, and *p*-adjust < 0.05 was considered to indicate statistically significant enrichment.

## 3. Results

### 3.1. Subcutaneous Fat Thickness of Castrated and Common Male Yaks

**Figure 1:**
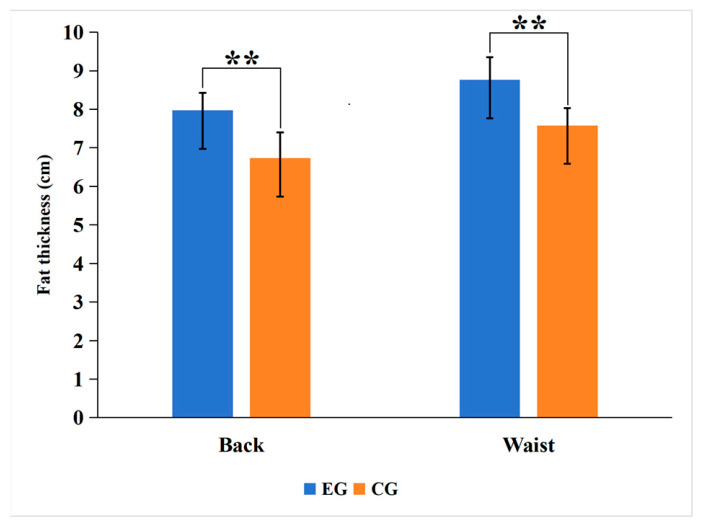
Bar chart for subcutaneous fat thickness in castrated male yaks and common male yaks. Two asterisks represented *p* < 0.01; EG, CG represented experimental group (castrated male yaks), control group (common male yaks), respectively.

The comparison of fat thickness in castrated and common male yaks is shown in Figure 1. The subcutaneous fat thickness in the back of EG yaks was 7.97 ± 0.45 mm, which was thicker than the value 6.73 ± 0.67 mm in the back of CG yaks (*p* < 0.01); the subcutaneous fat thickness in the waist of EG yaks was 8.77 ± 0.59 mm, which was thicker than the value 7.58 ± 0.45 mm in the back of CG yaks (*p* < 0.01). Therefore, it was inferred that the subcutaneous fat thickness in EG yaks was thicker than the value in CG yaks, and the results showed that the capacity of fat deposition in male yaks after being castrated was stronger.

### 3.2. Differentially Expressed Genes (DEGs) in Subcutaneous Fat Between Castrated and Common Male Yaks

**Figure 2:**
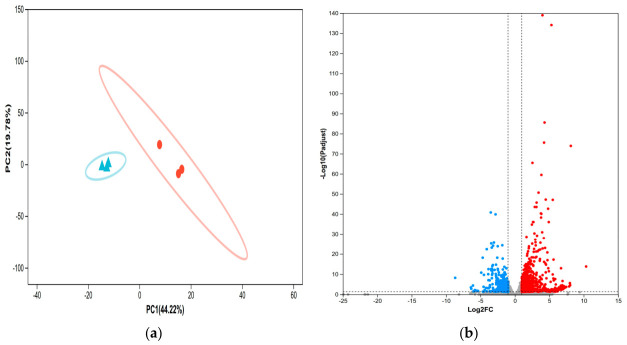
Comparison of gene expression in the subcutaneous fat of yaks between EG and CG. (**a**) Score plot of principal component analysis (PCA) for the gene expression in subcutaneous fat from EG and CG yaks. The horizontal axis indicated the contribution of the first principal component (PC1) to sample separation, and the vertical axis indicated the contribution of the second principal component (PC2) to sample separation. The circle represented the fat samples from CG, and the triangle represented the fat samples from EG. (**b**) Volcano plot of gene expression in yak subcutaneous fat (EG vs. CG). The horizontal axis represented fold change (FC) in gene expression between two groups, and the vertical axis represented the *p*-adjust value for the difference in gene expression level. A larger −log_10_ *p* adjust value indicated a more significant difference. Red dots indicated genes that were up-regulated in EG, blue dots indicated genes that were down-regulated in EG, and gray dots indicated genes with no significant difference in expression between the two groups.

Approximately 42.25 Gb of raw data was generated from yak fat samples. After strict quality control, a total of 40.64 Gb clean data was obtained, and the data size per sample was above 5.91 Gb. The percentage of Q30 base sequences exceeded 95.93%. After alignment with the yak reference genome, the alignment rate ranged from 90.38 to 91.88%. Therefore, the mRNA sequencing data from yak fat samples in this study was accurate and reliable. Score plot of PCA for the expression level of genes in the subcutaneous fat of EG and CG yaks is presented in Figure 2a. Samples from the two groups were distributed in two distinct areas, while samples within the same group clustered together, indicating high biological reproducibility within each group. These results suggest that castration substantially affects gene expression in yak subcutaneous fat. A total of 1416 DEGs were identified in yak subcutaneous fat between the EG and CG, and information on DEGs is shown in Table S2. Among these, 927 DEGs were up-regulated in the subcutaneous fat of EG yaks, whereas 468 DEGs were down-regulated (Figure 2b).

### 3.3. Gene Ontology (GO) Terms and Kyoto Encyclopedia of Genes and Genomes (KEGG) Pathways for DEGs Enrichment

**Figure 3:**
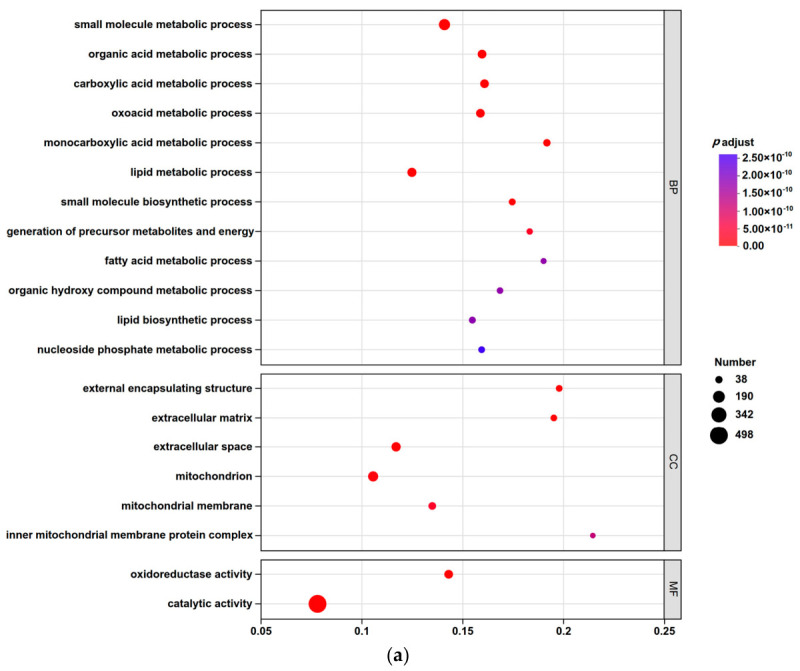

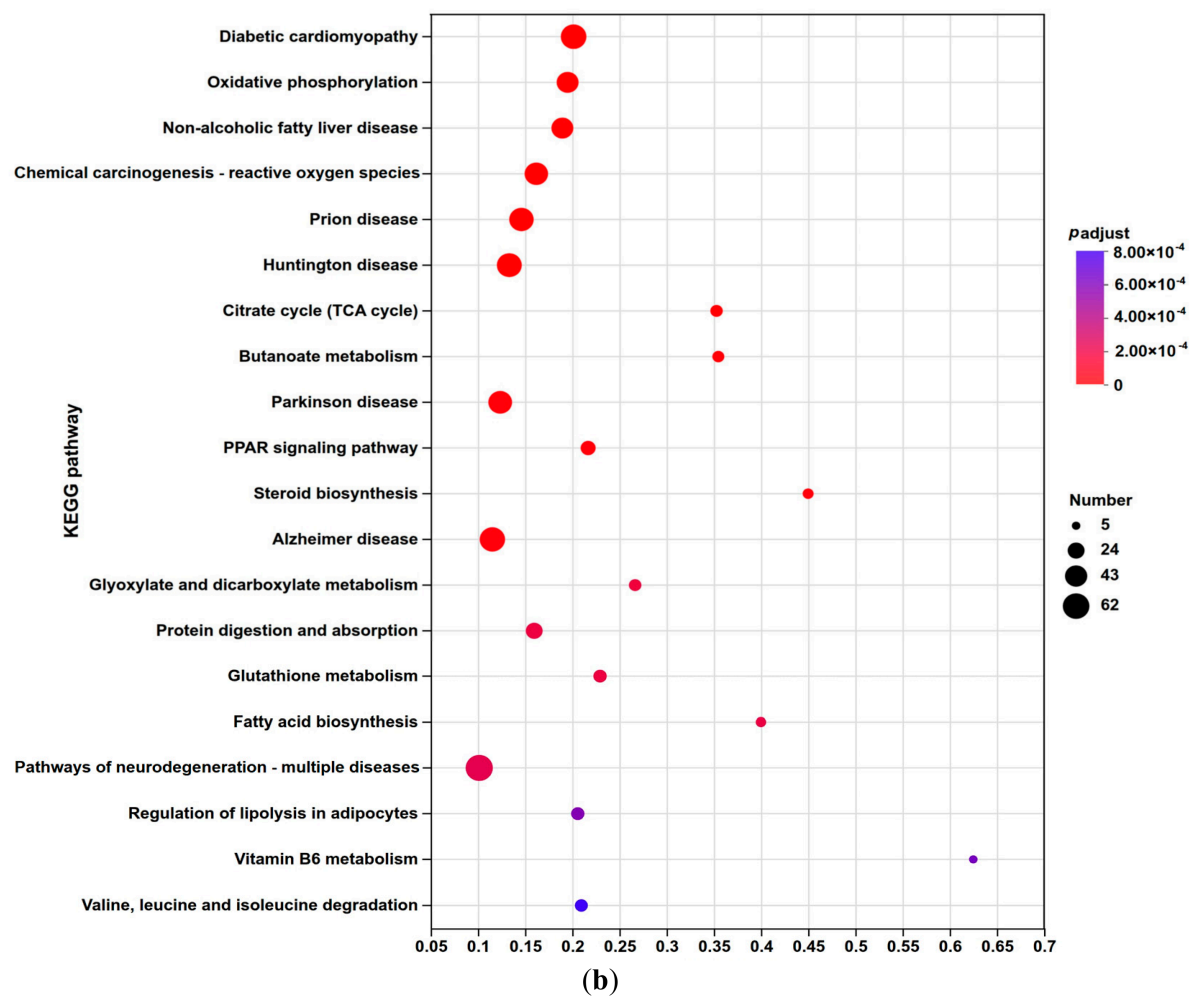
Biological function analysis of differentially expressed genes (DEGs). (**a**) Bubble diagram of the top 20 gene ontology (GO) terms for DEG enrichment. The horizontal axis represented the rich factor, and the vertical axis represented the GO term. Each bubble represented a GO Term. Bubble size was proportional to the number of DEGs enriched in the GO term, and bubble color indicated the enrichment significance of the GO term. (**b**) Bubble diagram of the top 20 significantly enriched Kyoto encyclopedia of genes and genome (KEGG) pathways for DEGs. The vertical axis represented the KEGG pathway, and the horizontal axis represented the richness factor. A larger richness factor indicated a greater enrichment degree.

The biological function of DEGs was explored through GO and KEGG enrichment analyses. The bar diagram of the top 20 significantly enriched GO items is presented in Figure 3a. These GO terms closely related to fat deposition included small molecule metabolic process, organic acid metabolic process, carboxylic acid metabolic process, oxoacid metabolic process, monocarboxylic acid metabolic process, oxidoreductase activity, extracellular matrix, catalytic activity, lipid metabolic process, extracellular space, mitochondrion, mitochondrial membrane, inner mitochondrial membrane protein complex, fatty acid metabolic process, and lipid biosynthetic process. The bubble diagram of the top 20 significantly enriched KEGG pathways is presented in Figure 3b. Pathways closely related to fat deposition mainly included oxidative phosphorylation, citrate cycle, butanoate metabolism, PPAR signaling pathway, steroid biosynthesis, glyoxylate and dicarboxylate metabolism, fatty acid biosynthesis, regulation of lipolysis in adipocytes, propanoate metabolism, pyruvate metabolism, and fatty acid degradation.

### 3.4. Differential Metabolites (DMs) in Subcutaneous Fat Between Castrated and Common Male Yaks

**Figure 4:**
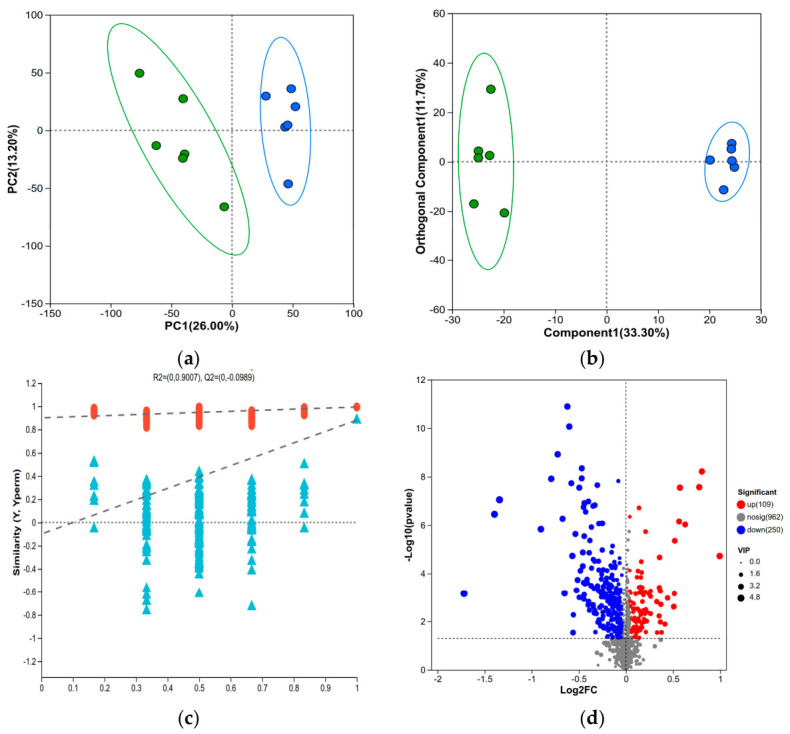
Metabolite profiles in yak subcutaneous fat of EG and CG. (**a**) Score plot of PCA for the metabolites in yak subcutaneous fat. The horizontal axis represented the contribution of PC1 to sample distinction, and the vertical axis represented the contribution of PC2 to sample distinction. Green circle: CG samples; blue circle: EG samples. (**b**) Score plot of orthogonal partial least squares-discriminant analysis (OPLS-DA) for the metabolites in yak subcutaneous fat of EG and CG. (**c**) Permutation testing for OPLS-DA mode validation. The horizontal axis represented the permutation retention degree, and the vertical axis represented the values of R^2^ (red dots) and Q^2^ (blue triangles). Two dashed lines indicated the regression lines for R^2^ and Q^2^, respectively. (**d**) Volcano plot of metabolites abundance in yak subcutaneous fat (EG vs. CG). Horizontal axis represented the value of log_2_ FC, and vertical axis represented the value of −log_10_ *p*. Each point represented a metabolite, and point size indicated VIP value. Red point: metabolites significantly up-regulated in EG; blue point: metabolites significantly down-regulated in EG; gray points: metabolites with no significant difference between EG and CG.

A total of 1372 metabolites were detected in yak fat samples. PCA for the metabolomics data revealed a clear separation between EG and CG (Figure 4a). Furthermore, the score plots of OPLS-DA showed that samples within each group clustered together, while there were significant differences in samples between EG and CG (Figure 4b), indicating the castration induced substantial metabolic changes in yak fat. The reliability of the OPLS-DA model was evaluated using a 7-fold cross-validation method (Figure 4c). The order of the categorical variable Y was randomly changed, and 200 permutations were conducted to establish corresponding OPLS-DA models. It was found that the R^2^ value of the original mode, 0.9077, was close to 1, which indicated the OPLS-DA model accurately fitted the actual data. The Q^2^ value −0.0989 was close to 0, which indicated the model with no overfitting and good stability adequately explained the differences between EG and CG. A total of 359 DMs were identified in yak subcutaneous fat between EG and CG (Figure 4d), and the information on DMs is shown in Table S3. Among these, 109 DMs were up-regulated, and 250 DMs were down-regulated in the subcutaneous fat of EG yaks.

### 3.5. KEGG Pathways for DM Enrichment

**Figure 5:**
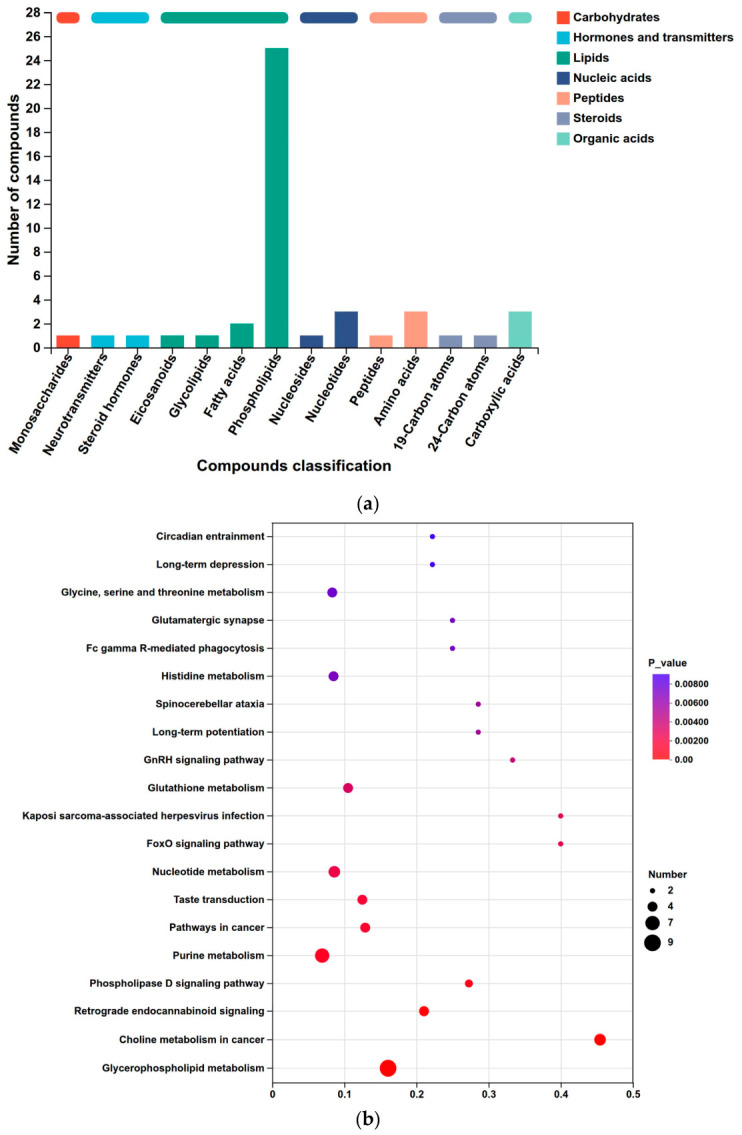
KEGG enrichment for DMs in yak fat between CG and EG. (**a**) KEGG classification of DMs. The vertical axis represented the secondary classification category in the KEGG database, and the horizontal axis represented the number of DMs annotated to each category. (**b**) Bubble diagram of the top 20 KEGG pathways for DM enrichment. The horizontal axis represented enrichment rate, and the vertical axis represented KEGG pathways. Bubble size represented the number of DMs enriched in each pathway, and bubble color indicated the significance of enrichment.

The classification statistics of DMs based on the KEGG database are presented in Figure 5a, and most of the DMs belonged to phospholipids. These DMs were enriched in a total of 176 KEGG pathways, and the bubble diagram of the top 20 KEGG pathways for DM enrichment is displayed in Figure 5b. The KEGG pathways involved in fat deposition mainly included glycerophospholipid metabolism, FoxO signaling pathway, pantothenate and CoA biosynthesis, calcium signaling pathway, sphingolipid signaling pathway, insulin resistance, butanoate metabolism, and phospholipase D signaling pathway.

### 3.6. Fatty Acids Content in Subcutaneous Fat of Castrated and Common Male Yaks

The score plots of PCA and OPLS-DA for fatty acids showed the fat samples in each group clustered together (Figures S1 and S2), while clear separation was observed between the EG and CG, indicating that castration induced significant alterations in the fatty acid profile of male yak fat. A total of 30, 28 fatty acids were detected in the subcutaneous fat of EG and CG yaks, respectively (Table 1). Significant differences between EG and CG were observed for 21 fatty acids, including C8:0, C15:0, *cis9*-C16:1, C17:0, *cis*-C17:1, C18:0, *trans9*-C18:1, *trans9*,*trans12*-C18:2, *cis9*,*cis12*-C18:2, *cis6*,*cis9*,*cis12*-C18:3, *cis9*,*cis12*,*cis15*-C18:3, C20:0, *cis11*,*cis14*-C20:2, C21:0, *cis5*,*cis8*,*cis11*,*cis14*-C20:4, *cis11*,*cis14*,*cis17*-C20:3, C22:0, *cis5*,*cis8*,*cis11*,*cis14*,*cis17*-C20:5, *cis13*-C22:1, C23:0 and *cis15*-C24:1 (*p* < 0.05). Among these, the content of C8:0, C15:0, C17:0, *cis*-C17:1, C18:0, *trans9*-C18:1, *trans9*,*trans12*-C18:2, *cis11*,*cis14*-C20:2, *cis9*,*cis12*-C18:2, *cis6*,*cis9*,*cis12*-C18:3, *cis9*,*cis12*,*cis15*-C18:3, C20:0, C21:0, *cis11*,*cis14*,*cis17*-C20:3, C22:0, C23:0, *cis15*-C24:1 and *cis5*,*cis8*,*cis11*,*cis14*,*cis17*-C20:5 was higher in EG than in CG (*p* < 0.05), whereas the content of only *cis9*-C16:1, *cis5*,*cis8*,*cis11*,*cis14*-C20:4 and *cis13*-C22:1 was lower in EG than in CG (*p* < 0.05). Moreover, the content of total fatty acids was significantly higher in EG than in CG (*p* < 0.05), and the value of ΣPUFAs was significantly higher in EG than in CG too (*p* < 0.05).

### 3.7. Validation Result for Transcriptome

**Figure 6:**
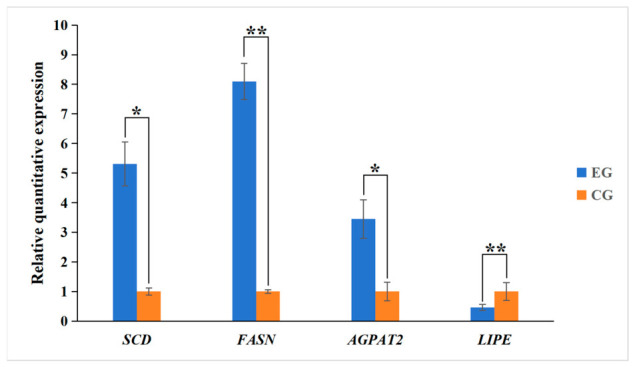
Quantitative result for the expression level of selected genes in yak subcutaneous fat from EG and CG by RT-qPCR. One asterisk represented *p* < 0.05, and two asterisks represented *p* < 0.01.

*RT-qPCR* analysis revealed significant differences in the expression level of the selected four DEGs between the EG and CG. The expression level of *SCD*, *FASN*, and *AGPAT2* genes was up-regulated in yak subcutaneous fat of EG, whereas the expression level of the *LIPE* gene was down-regulated in yak subcutaneous fat of EG (Figure 6). All four DEGs exhibited similar expression patterns in comparison to the mRNA-seq data, which indicated the reliability of the mRNA-seq results for transcriptome analysis.

### 3.8. Integrative Analysis for Transcriptome, Metabolome, Fatty Acids, and Fat Thickness

#### 3.8.1. Results of Integrative Analysis for Transcriptome and Metabolome

**Figure 7:**
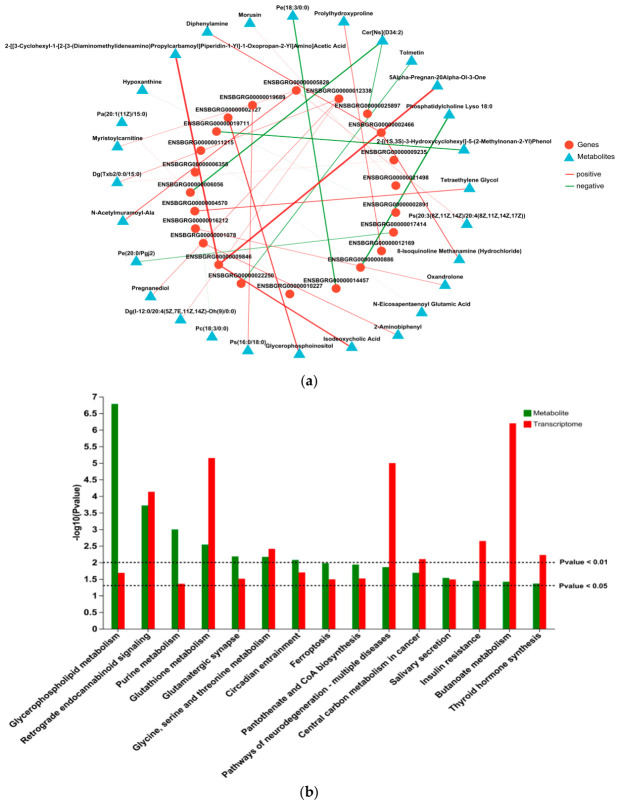
Correlation of DEGs, DMs, fatty acids, and fat thickness. (**a**) Network diagram of the top 30 correlations between DMs and DEGs. Triangles, circles, and lines represented metabolites, genes, and correlation coefficients, respectively. Red lines indicated positive correlations, and green lines indicated negative correlations. Line thickness was proportional to the absolute value of the correlation coefficient (a thicker line indicated a stronger correlation). (**b**) Bar diagram of shared KEGG pathways enriched by both DEGs and DMs. Horizontal axis represented KEGG pathways, and vertical axis represented the −log_10_*p* value. Red bars represented the enrichment results of DEGs, and green bars indicated the enrichment results of DMs.

A network diagram showing the top 30 correlation coefficients between DMs and DEGs is presented in Figure 7a, and the bar diagram of shared KEGG pathways enriched by DEGs and DMs is shown in Figure 7b. Integrative analysis revealed these pathways, including glycerophospholipid metabolism, pantothenate and CoA biosynthesis, insulin resistance, butanoate metabolism, fatty acid biosynthesis, PPAR signaling pathway, citrate cycle, cholesterol metabolism, and steroid biosynthesis, played important roles in the effect of castration on the fat deposition in male yaks after castration. It was preliminarily inferred that *FASN*, *ACACA*, *ACACB*, *ACSL4*, *AGPAT2*, *ACLY*, *ACSL5*, *CK2*, *SCD*, *LPL*, *ME1*, *FADS2*, *ACOX2*, *SREBF1*, *ATP2A1*, *GSK3B*, *SLC2A4*, *SLC27A3*, *BDH1*, and *ACADS* were the key regulatory genes for lipid metabolism in the subcutaneous fat of castrated male yaks. Furthermore, the metabolites Ps(20:3(8Z,11Z,14Z)/20:4(8Z,11Z,14Z,17Z)), Dg(11M3/13M5/0:0), Pa(20:1(11Z)/15:0), Dg(15:0/20:3(5Z,8-Z,11Z)/0:0), O-acetylcarnitine, pantetheine, panthenol, maleic acid, L-glutamic acid, and 4-hydroxybutanoic acid were identified as crucial in this process.

#### 3.8.2. Correlation Results Between Important DEGs and Fatty Acids, Fat Thickness

**Figure 8:**
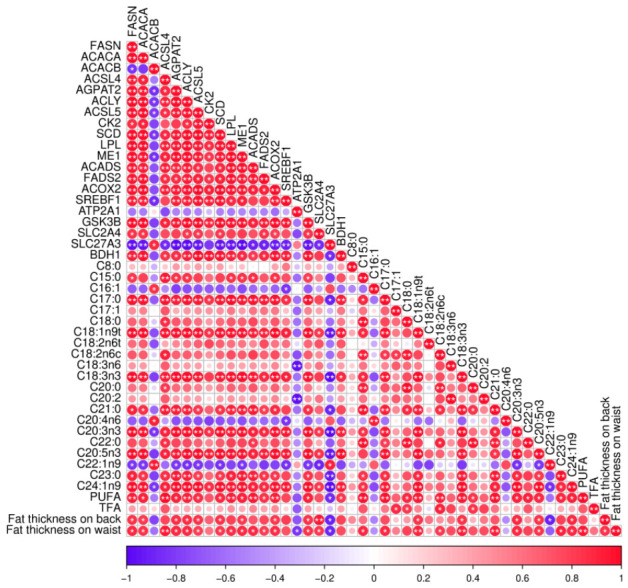
Correlation matrix of important DEGs, fatty acids, and fat thickness in yaks. * showed *p* < 0.05, ** showed *p* < 0.01. Circle with no * or ** showed *p* > 0.05. Red and blue represented positive and negative correlation, respectively. The deeper the color was, the higher the correlation was.

The correlation among important DEGs, fatty acids, and subcutaneous fat thickness is presented in Figure 8. Fat thickness both on the yak back and waist showed a high positive correlation with the expression of *FASN*, *ACACA*, *AGPAT2*, *ACLY*, *ACSL5*, *SCD*, *ME1*, *ACOX2*, *GSK3B*, and *SLC2A4* genes (*p* < 0.05, r > 0.8), whereas it was in a high negative correlation with the expression of the *SLC27A3* gene (*p* < 0.05, r > 0.8). Similarly, PUFA content in yak subcutaneous fat was in a high positive correlation with the expression of *FASN*, *ACACA*, *ACSL4*, *AGPAT2*, *ACLY*, *ACSL5*, *SCD*, *LPL*, *ME1*, *ACADS*, *FADS2*, *ACOX2*, *GSK3B*, and *BDH1* genes (*p* < 0.05, r > 0.8), whereas it was in a high negative correlation with the expression of the *SLC27A3* gene (*p* < 0.05, r > 0.8).

## 4. Discussion

Castrated yaks exhibited significantly greater thickness of subcutaneous fat than common male yaks, which initially demonstrated that castration enhanced the fat deposition in yaks. Important KEGG pathways enriched by DEGs and DMs were primarily associated with lipid metabolism, energy homeostasis, and insulin signaling, and it was inferred that castration in yak promoted lipid synthesis, increased substrate availability, and activated adipogenic transcription factors. Furthermore, the effect of castration on the fat deposition in yaks was realized by the coordinated regulation based on fatty acid biosynthesis, PPAR signaling, and energy metabolism pathways. PPARs are the master regulators of adipogenesis and lipid storage, and their activation is frequently associated with adipose tissue expansion in cattle [17,18]. Therefore, castration may activate the PPAR signaling in yak adipose tissue, thereby promoting fatty acid synthesis and lipid accumulation. Insulin resistance is often accompanied by localized insulin signaling alterations without systemic metabolic dysfunction in cows [19] and may reflect a state of nutrient surplus and adipocyte hypertrophy in these castrated yaks. These enriched KEGG pathways, including fatty acid biosynthesis, glycerophospholipid metabolism, cholesterol metabolism, and steroid biosynthesis, suggested that castration enhanced de novo lipogenesis and membrane lipid remodeling in adipose tissue of male yak. Diacylglycerol is a precursor for triglyceride synthesis [20]. Dg(15:0/20:3) and Dg(11M3/13M5) served as substrates for triglyceride accumulation, ultimately contributing to increasing fat thickness in yaks. Phosphatidylserine and phosphatidic acid are closely associated with the formation of the cell membrane phospholipid bilayer [21,22], so Ps(20:3/20:4) and Pa(20:1/15:0) played key roles in the remodeling of adipocyte plasma membrane in male yak after castration. Castration leads to a reduction in circulating testosterone in cattle [23], which promotes the expression of lipogenic enzymes. Cholesterol metabolism and steroid biosynthesis are closely linked to the balance of sex hormones such as testosterone [24], and the enrichment of these two pathways may reflect the relief of androgen-mediated inhibition for fat deposition in yak. Meanwhile, the enrichment of the TCA cycle, butanoate metabolism, and pantothenate and CoA biosynthesis collectively indicated a coordinated enhancement of energy metabolism in yak adipose tissue. CoA biosynthesis is essential for fatty acid activation and elongation, thereby further supporting enhanced fat deposition [25]. The TCA cycle provides both ATP and metabolic intermediates for lipogenesis [26], while butanoate metabolism, which links to the short-chain fatty acids derived from rumen microbiota, also contributes to adipogenesis [27]. Furthermore, O-acetylcarnitine [28], pantetheine, panthenol, and 4-hydroxybutanoic acid [29] are involved in mitochondrial fatty acid oxidation and CoA homeostasis. O-acetylcarnitine facilitates the transport of acetyl units from peroxisomes to mitochondria, while pantetheine and panthenol serve as precursors for CoA synthesis. Four-hydroxybutanoic acid is an intermediate for energy metabolism in the cycle and is also associated with partial inhibition of fatty acid oxidation [30]. Maleic acid is also an intermediate in the TCA cycle, and L-glutamic acid is involved in anaplerosis of the TCA cycle [31]. Collectively, these observations suggested O-acetylcarnitine, pantetheine, panthenol, 4-hydroxybutanoic acid, maleic acid, and L-glutamic acid may function as important intermediates providing carbon or affecting fatty acid oxidation to support enhanced lipid synthesis in the adipose tissue of castrated male yaks.

Fat deposition in yak is regulated by specific expression of genes [32], which primarily control fatty acid transport, de novo fatty acid synthesis, fatty acid hydrogenation or dehydrogenation, lipid synthesis, lipolysis, and the number of lipid transport carriers, ultimately influencing the amount of lipid stored in tissues and organs [33]. There are interactions between glucose metabolism and lipid metabolism in beef cattle [34], and the substrate for fat deposition in yaks mainly includes volatile fatty acids and glucose. Castration induces systemic transcriptional reprogramming in yak adipose tissue. Positive correlation between the expression level of *FASN*, *ACACA*, *AGPAT2*, *ACLY*, *ACSL5*, *SCD*, *ME1*, *ACOX2*, *GSK3B*, *SLC2A4* genes and the subcutaneous fat thickness in yaks was identified, and these genes function encompassed the regulation of de novo lipogenesis, fatty acid activation and desaturation, and insulin sensitivity. *FASN* [35], *SCD* [36], *ACLY* [37], and *ACACA* [38] genes are the crucial control genes for fatty acid synthesis in bovines. Furthermore, the esterification of fatty acids into triglycerides is crucial for fat deposition, and both *ACSL5* [39] and *AGPAT2* [18] genes are key regulatory genes for triglyceride biosynthesis in bovines. The *GSK3B* gene is typically associated with insulin signaling inhibition in bovine [40], and the *SLC2A4* gene regulates glucose transmembrane transport in cows [41], so *GSK3B* and *SLC2A4* genes may regulate the process of glucose-derived carbons translating into lipids. It was inferred that the stronger ability of fat deposition in castrated yaks was mainly regulated by *FASN*, *ACACA*, *AGPAT2*, *ACLY*, *ACSL5*, *SCD*, *GSK3B*, and *SLC2A4* genes. PUFAs possess various physiological functions in the human body, including reducing cholesterol and triglycerides in the blood, lowering blood viscosity, enhancing the activity of brain cells, strengthening memory and thinking ability [42,43]. Notably, cattle could only obtain PUFAs from feed and could not synthesize PUFAs in vivo [44]. The finding that the PUFA content in castrated male yaks was higher than that in common male yaks was of great significance and deserved attention. Dietary PUFAs are primarily transported in chylomicrons. The *LPL* gene is known to positively regulate PUFA release from the lipoproteins [45], and its up-regulated expression promotes PUFA uptake from blood and storage in adipose tissue. Up-regulated expression of the *FADS2* gene promotes the biosynthesis of long-chain PUFAs andΔ6-desaturation of linoleic acid (18:2n-6) and α-linolenic acid (18:3n-3) in bovine [46,47]. Concurrently, the *ACSL4* gene regulates the conversion of PUFAs into their CoA esters [48,49], which enriches PUFA content in adipose tissue. PUFA content in yak fat was highly positively correlated with the expression of *ACSL4*, *LPL*, and *FADS2* genes. Therefore, it was inferred that *FADS2*, *LPL*, and *ACSL4* genes were closely related to PUFA content in yak fat tissue, and the higher PUFA content in castrated yak fat was positively regulated by these genes.

## 5. Conclusions

Castration enhances the capacity of fat deposition and alters fatty acid content, particularly increasing PUFA content in the adipose tissue of male yaks. The effect of castration on fat deposition in male yaks was primarily mediated through the regulation of the PPAR signaling pathway, citrate cycle, and insulin resistance. Consequently, the downstream signaling pathways, including glycerophospholipid metabolism, pantothenate and CoA biosynthesis, butanoate metabolism, and fatty acid biosynthesis, were also modulated. Data suggests that *FASN*, *ACACA*, *AGPAT2*, *ACLY*, *ACSL5*, *SCD*, *GSK3B*, and *SLC2A4* genes may serve as the crucial control genes for fat deposition in castrated male yaks, and that *FADS2*, *LPL*, and *ACSL4* genes are the crucial control genes for higher PUFAs content in adipose tissue of castrated yaks. The additional functional studies will be conducted to determine the specific role of each gene in regulating each function in the future.

## Figures and Tables

**Table 1:** Content of fatty acids in yak subcutaneous fat of the experimental group (EG) and the control group (CG).

Index	EG (Mean ± RSD, μg/g)	CG (Mean ± RSD, μg/g)	*p*
C6:0	ND	ND	-
C8:0	4.72 ± 0.31	3.61 ± 0.43	<0.001
C10:0	15.32 ± 3.68	19.73 ± 5.06	0.15
C11:0	2.79 ± 0.15	2.76 ± 0.05	0.71
C12:0	14.85 ± 1.10	17.55 ± 4.87	0.25
C13:0	8.88 ± 0.92	7.63 ± 1.62	0.16
C14:0	475.92 ± 50.30	551.93 ± 141.51	0.28
*cis9*-C14:1	34.67 ± 8.64	54.48 ± 18.66	0.06
C15:0	252.73 ± 33.52	133.29 ± 40.28	<0.001
*cis*-C15:1	11.63 ± 0.86	13.06 ± 2.24	0.21
C16:0	5739.07 ± 426.61	6086.17 ± 1332.91	0.59
*cis9*-C16:1	490.76 ± 120.14	835.59 ± 263.26	0.02
C17:0	1048.86 ± 135.19	293.84 ± 75.34	<0.001
*cis*-C17:1	205.91 ± 33.61	145.71 ± 37.56	0.02
C18:0	11,045.30 ± 1264.66	8030.58 ± 20,258.47	0.02
*trans9*-C18:1	3649.16 ± 289.96	801.53 ± 208.08	<0.001
*cis9*-C18:1	9622.31 ± 1360.76	10,727.27 ± 2631.07	0.42
*trans9*,*trans12*-C18:2	76.97 ± 6.41	64.70 ± 6.34	0.01
*cis9*,*cis12*-C18:2	822.63 ± 129.00	492.20 ± 149.65	<0.001
*cis6*,*cis9*,*cis12*-C18:3	41.86 ± 1.68	36.26 ± 1.22	<0.001
*cis9*,*cis12*,*cis15*-C18:3	444.99 ± 72.89	65.28 ± 10.22	<0.001
C20:0	172.83 ± 20.92	110.04 ± 44.76	0.02
*cis13*-C20:1	ND	ND	-
*cis11*,*cis14*-C20:2	39.60 ± 2.68	35.96 ± 1.09	0.02
C21:0	58.65 ± 4.59	42.58 ± 2.54	<0.001
*cis8*,*cis11*,*cis14*-C20:3	ND	ND	-
*cis5*,*cis8*,*cis11*,*cis14*-C20:4	51.79 ± 3.07	57.47 ± 4.36	0.04
*cis11*,*cis14*,*cis17*-C20:3	43.07 ± 2.21	ND	<0.001
C22:0	76.61 ± 9.15	56.67 ± 9.37	<0.001
*cis5*,*cis8*,*cis11*,*cis14*,*cis17*-C20:5	35.02 ± 3.13	ND	<0.001
*cis13*-C22:1	ND	69.41 ± 26.16	<0.001
*cis13*,*cis16*-C22:2	ND	ND	-
C23:0	22.40 ± 4.73	10.15 ± 1.53	<0.001
C24:0	47.05 ± 1.83	45.70 ± 1.43	0.22
*cis15*-C24:1	61.72 ± 4.88	ND	<0.001
*cis4*,*cis7*,*cis10*,*cis13*,*cis16*,*cis19*-C22:6	ND	ND	-
ΣSFAs	18,985.98 ± 1353.07	15,412.22 ± 3564.82	0.06
ΣUFAs	15,622.51 ± 1769.89	13,398.91 ± 3218.55	0.21
ΣMUFAs	14,066.58 ± 1670.98	12,647.04 ± 3090.32	0.39
ΣPUFAs	1555.93 ± 208.89	751.87 ± 163.78	<0.001
Total FA	50,231.00 ± 4184.09	42,210.04 ± 3800.64	0.01

ΣMUFAs: total monounsaturated fatty acids; ΣSFAs: total saturated fatty acids; ΣPUFAs: total polyunsaturated fatty acids; ΣUFAs: total unsaturated fatty acids; FA: fatty acids; RSD: relative standard deviation; ND: no detection.

## Data Availability

The datasets generated for this study can be found in the Sequence Read Archive (https://www.ncbi.nlm.nih.gov/sra, accessed on 21 April 2026) at NCBI, with the BioProject ID PRJNA1455729.

## Supplementary Materials

The following supporting information can be downloaded at: https://www.mdpi.com/article/10.3390/ani16121825/s1, Table S1: Information of primer for the four selected DEGs in the yak subcutaneous fat of two groups; Table S2: Information of DEGs in the yak subcutaneous fat between EG and CG; Table S3: Information of DMs in the yak subcutaneous fat of EG and CG; Figure S1: Score plots of PCA for the fatty acids in the yak subcutaneous fat of EG and CG. Figure S2: Score plots of OPLS-DA for the fatty acids in the yak subcutaneous fat of EG and CG.

## References

[1] Stafford K.J., Mellor D.J. (2005). The welfare significance of the castration of cattle: A review. N. Z. Vet. J..

[2] Roberts S.L., Powell J.G., Hughes H.D., Richeson J.T. (2018). Effect of castration method and analgesia on inflammation, behavior, growth performance, and carcass traits in feedlot cattle. J. Anim. Sci..

[3] Bretschneider G. (2005). Effects of age and method of castration on performance and stress response of beef male cattle: A review. Livest. Prod. Sci..

[4] Siel D., Huenchullán P.R., Vidal S., Valdés A., Sáenz L. (2024). Improving Beef Cattle Production: Safety and Effectiveness of New Immunocastration Vaccine. Animals.

[5] Liu M., Su N., Ma Z., Chen W., Zhang Y., Yan X., Liu W. (2026). Meat Quality Differences Correlated with Rumen Microbiota and Lipid Metabolism in Beef Cattle vs. Castrated Cattle. Int. J. Mol. Sci..

[6] Cara J.R.F., de Araújo T.L.A.C., Néves A.P., Latta K.I., Pereira M.W.F., de Oliveira Menezes G.R., Feijó G.L.D., de Nadai Bonin Gomes M., da Costa Gomes R. (2025). Castration procedures and feed management in the finishing phase affect the performance, carcass traits, and meat quality of grazing steers. Trop. Anim. Health Prod..

[7] Reis I.A., Baldassini W.A., Ramírez-Zamudio G.D., de Farias I.M.S.C., Chiaratti M.R., Pereira Junior S., Nociti R.P., Carvalho P.H.V., Curi R.A., Pereira G.L. (2024). Muscle tissue transcriptome of F1 Angus-Nellore bulls and steers feedlot finished: Impacts on intramuscular fat deposition. BMC Genom..

[8] Shi J., Li Z., Jia L., Ma Y., Huang Y., He P., Ran T., Liu W., Zhang W., Cheng Q. (2024). Castration alters the ileum microbiota of Holstein bulls and promotes beef flavor compounds. BMC Genom..

[9] Li Z., Shi J., Lei Y., Wu J., Zhang R., Zhang X., Jia L., Wang Y., Ma Y., He P. (2022). Castration alters the cecal microbiota and inhibits growth in Holstein cattle. J. Anim. Sci..

[10] Jiang H., Chai Z.X., Chen X.Y., Zhang C.F., Zhu Y., Ji Q.M., Xin J.W. (2024). Yak genome database: A multi-omics analysis platform. BMC Genom..

[11] Qiu Q., Zhang G., Ma T., Qian W., Wang J., Ye Z., Cao C., Hu Q., Kim J., Larkin D.M. (2012). The yak genome and adaptation to life at high altitude. Nat. Genet..

[12] Zhang J., Mamet T., Guo Y., Li C., Yang J. (2023). Yak milk promotes renal calcium reabsorption in mice with osteoporosis via the regulation of TRPV5. J. Dairy Sci..

[13] He Z., Wang X., Qi Y., Zhu C., Zhao Z., Zhang X., Liu X., Li S., Zhao F., Wang J. (2023). Long-stranded non-coding RNAs temporal-specific expression profiles reveal longissimus dorsi muscle development and intramuscular fat deposition in Tianzhu white yak. J. Anim. Sci..

[14] Xiong L., Pei J., Chu M., Wu X., Kalwar Q., Yan P., Guo X. (2021). Fat Deposition in the Muscle of Female and Male Yak and the Correlation of Yak Meat Quality with Fat. Animals.

[15] Xu F., Wang H., Qin C., Yue B., Yang Y., Wang J., Zhong J., Wang H. (2024). Combined Multi-Omics Analysis Reveals the Potential Role of ACADS in Yak Intramuscular Fat Deposition. Int. J. Mol. Sci..

[16] Ma J., Wang H., Yin Y., Meng X., Huang C., Xu F., Qin C., Wang H., Chai Z., Kangzhu Y. (2025). Role of super-enhancer-associated miRNAs and histone modifications in intramuscular fat deposition in yak. Int. J. Biol. Macromol..

[17] Lim D., Chai H.H., Lee S.H., Cho Y.M., Choi J.W., Kim N.K. (2015). Gene Expression Patterns Associated with Peroxisome Proliferator-activated Receptor (PPAR) Signaling in the Longissimus dorsi of Hanwoo (Korean Cattle). Asian-Australas. J. Anim. Sci..

[18] Tan Z., Pokhrel B., Zhou Z., Jiang H. (2025). Transcriptome analysis unveils multiple reasons behind delayed and slower deposition of intramuscular fat compared to subcutaneous fat in cattle. BMC Genom..

[19] Ghaffari M.H., Sadri H., Sauerwein H. (2023). Invited review: Assessment of body condition score and body fat reserves in relation to insulin sensitivity and metabolic phenotyping in dairy cows. J. Dairy Sci..

[20] Choi Y.M., Ajjaji D., Fleming K.D., Borbat P.P., Jenkins M.L., Moeller B.E., Fernando S., Bhatia S.R., Freed J.H., Burke J.E. (2023). Structural insights into perilipin 3 membrane association in response to diacylglycerol accumulation. Nat. Commun..

[21] Leventis P.A., Grinstein S. (2010). The distribution and function of phosphatidylserine in cellular membranes. Annu. Rev. Biophys..

[22] Kim S.C., Wang X. (2020). Phosphatidic acid: An emerging versatile class of cellular mediators. Essays Biochem..

[23] Ahn J.S., Kwon E.G., Lee H.J., Kim U.H., Won J.I., Jang S.S., Park B.K. (2023). Effect of short-term fattening period and castration method on productivity, serum testosterone, and economic efficacy in Hanwoo cattle. J. Anim. Sci. Technol..

[24] Zhu Z., Chung Y.M., Alyamani M., Dai Y., McCarty K.D., Roberts E., Sinha S., Li J., Li X., Gad E.M. (2025). A bypass gateway from cholesterol to sex steroid biosynthesis circumnavigates CYP17A1. Nat. Commun..

[25] Ohlrogge J., Browse J. (1995). Lipid biosynthesis. Plant Cell.

[26] Inigo M., Deja S., Burgess S.C. (2021). Ins and Outs of the TCA Cycle: The Central Role of Anaplerosis. Annu. Rev. Nutr..

[27] Cheng J., Zhang Y., Ge Y., Li W., Cao Y., Qu Y., Fu S., Liu J. (2020). Sodium butyrate promotes milk fat synthesis in bovine mammary epithelial cells via GPR41 and its downstream signalling pathways. Life Sci..

[28] Izzo L.T., Trefely S., Demetriadou C., Drummond J.M., Mizukami T., Kuprasertkul N., Farria A.T., Nguyen P.T.T., Murali N., Reich L. (2023). Acetylcarnitine shuttling links mitochondrial metabolism to histone acetylation and lipogenesis. Sci. Adv..

[29] Silva A.R., Ruschel C., Helegda C., Brusque A.M., Wannmacher C.M., Wajner M., Dustra-Filho C.S. (1999). Inhibition of rat brain lipid synthesis in vitro by 4-hydroxybutyric acid. Metab. Brain Dis..

[30] Brown G.K., Cromby C.H., Manning N.J., Pollitt R.J. (1987). Urinary organic acids in succinic semialdehyde dehydrogenase deficiency: Evidence of alpha-oxidation of 4-hydroxybutyric acid, interaction of succinic semialdehyde with pyruvate dehydrogenase and possible secondary inhibition of mitochondrial beta-oxidation. J. Inherit. Metab. Dis..

[31] Tapiero H., Mathé G., Couvreur P., Tew K.D. (2002). Glutamine and glutamate. Biomed. Pharmacother..

[32] Wang H., Zhong J., Zhang C., Chai Z., Cao H., Wang J., Zhu J., Wang J., Ji Q. (2020). The whole-transcriptome landscape of muscle and adipose tissues reveals the ceRNA regulation network related to intramuscular fat deposition in yak. BMC Genom..

[33] Lee J.N., Wang Y., Xu Y.O., Li Y.C., Tian F., Jiang M.F. (2017). Characterisation of gene expression related to milk fat synthesis in the mammary tissue of lactating yaks. J. Dairy Res..

[34] He T., Yi G., Li J., Wu Z., Guo Y., Sun F., Liu J., Tang C., Long S., Chen Z. (2023). Dietary Supplementation of Tannic Acid Promotes Performance of Beef Cattle via Alleviating Liver Lipid Peroxidation and Improving Glucose Metabolism and Rumen Fermentation. Antioxidants.

[35] Raza S.H.A., Gui L., Khan R., Schreurs N.M., Wu S., Mei C., Wang L., Ma X., Wei D., Guo H. (2018). Association between FASN gene polymorphisms ultrasound carcass traits and intramuscular fat in Qinchuan cattle. Gene.

[36] Gao Y.Y., Cheng G., Cheng Z.X., Bao C., Yamada T., Cao G.F., Bao S.Q., Schreurs N.M., Zan L.S., Tong B. (2022). Association of variants in FABP4, FASN, SCD, SREBP1 and TCAP genes with intramuscular fat, carcass traits and body size in Chinese Qinchuan cattle. Meat Sci..

[37] Li M.N., Guo X., Bao P.J., Wu X.Y., Ding X.Z., Chu M., Liang C.N., Yan P. (2016). Association of genetic variations in the ACLY gene with growth traits in Chinese beef cattle. Genet. Mol. Res..

[38] Singh A., Malla W.A., Kumar A., Jain A., Thakur M.S., Khare V., Tiwari S.P. (2023). Review: Genetic background of milk fatty acid synthesis in bovines. Trop. Anim. Health Prod..

[39] Yu X., Fang X., Xiao H., Zhao Z., Maak S., Wang M., Yang R. (2019). The effect of acyl-CoA synthetase long-chain family member 5 on triglyceride synthesis in bovine preadipocytes. Arch. Anim. Breed..

[40] Du Y., Guo J., Zhao Z., Qin L., Zhang J., Zhao Y., Peng Y. (2026). Bovine Preadipocyte-Derived Exosomal miR-26a Promotes Intramuscular Adipogenesis by Directly Targeting GSK3B. J. Agric. Food Chem..

[41] Weber C., Schäff C.T., Kautzsch U., Börner S., Erdmann S., Bruckmaier R.M., Röntgen M., Kuhla B., Hammon H.M. (2017). Variable liver fat concentration as a proxy for body fat mobilization postpartum has minor effects on insulin-induced changes in hepatic gene expression related to energy metabolism in dairy cows. J. Dairy Sci..

[42] Wiktorowska-Owczarek A., Berezińska M., Nowak J.Z. (2015). PUFAs: Structures, Metabolism and Functions. Adv. Clin. Exp. Med..

[43] Park Y., Watkins B.A. (2022). Dietary PUFAs and Exercise Dynamic Actions on Endocannabinoids in Brain: Consequences for Neural Plasticity and Neuroinflammation. Adv. Nutr..

[44] Cheng G., Fu C., Wang H., Adoligbe C., Wei S., Li S., Jiang B., Wang H., Zan L. (2015). Production of transgenic beef cattle rich in n-3 PUFAs by somatic cell nuclear transfer. Biotechnol. Lett..

[45] Saxena U., Witte L.D., Goldberg I.J. (1989). Release of endothelial cell lipoprotein lipase by plasma lipoproteins and free fatty acids. J. Biol. Chem..

[46] Ibeagha-Awemu E.M., Akwanji K.A., Beaudoin F., Zhao X. (2014). Associations between variants of FADS genes and omega-3 and omega-6 milk fatty acids of Canadian Holstein cows. BMC Genet..

[47] Fu Y., Jia R., Xu L., Su D., Li Y., Liu L., Ma Z., Sun D., Han B. (2022). Fatty acid desaturase 2 affects the milk-production traits in Chinese Holsteins. Anim. Genet..

[48] Wang Y., Hu M., Cao J., Wang F., Han J.R., Wu T.W., Li L., Yu J., Fan Y., Xie G. (2025). ACSL4 and polyunsaturated lipids support metastatic extravasation and colonization. Cell.

[49] Kuwata H., Hara S. (2019). Role of acyl-CoA synthetase ACSL4 in arachidonic acid metabolism. Prostaglandins Other Lipid Mediat..

