# Supplementary material for: Transcriptomics and Metabolomics Signatures of Fat Deposition Following Orchiectomy in Yak
Source: Animals (Basel). 2026 Jun 12;16(12):1825. doi: 10.3390/ani16121825 (PMC13296150; doi:10.3390/ani16121825)
Supplement: Supplementary file 1 [file animals-16-01825-s001.zip › Table S1.pdf]

Table S1 Information of primer for the 4 selected differentially expressed genes (DEGs) in yak subcutaneous fat

Gene ID	Gene Symbol	Forward primer (5->3)	Reverse primer (5->3)	Product length (bp)	Tm (℃)
ENSBGRG00000024230	*SCD*	TACCACGTTCTTCATTGATTGC	TGTAGCTTTCCTCTCCAGTT	113	60
ENSBGRG00000005349	*FASN*	CCTACACTCAGAGCTACCG	TGCATGAAGAAGCACATGG	82	60
ENSBGRG00000025897	*AGPAT2*	GACATGATGGGCCTCATGGA	CTGCCGGTTGATGAAGAGGA	126	60
ENSBGRG00000018426	*LIPE*	CTTCTTCGAGGGTGATGAG	CGGGTGTGAACTGGAAAC	107	60
