# Supplementary figures and images for: Transcriptomics and Metabolomics Signatures of Fat Deposition Following Orchiectomy in Yak
Source: Animals (Basel). 2026 Jun 12;16(12):1825. doi: 10.3390/ani16121825 (PMC13296150; doi:10.3390/ani16121825)

# OPLSDA Scores Plot

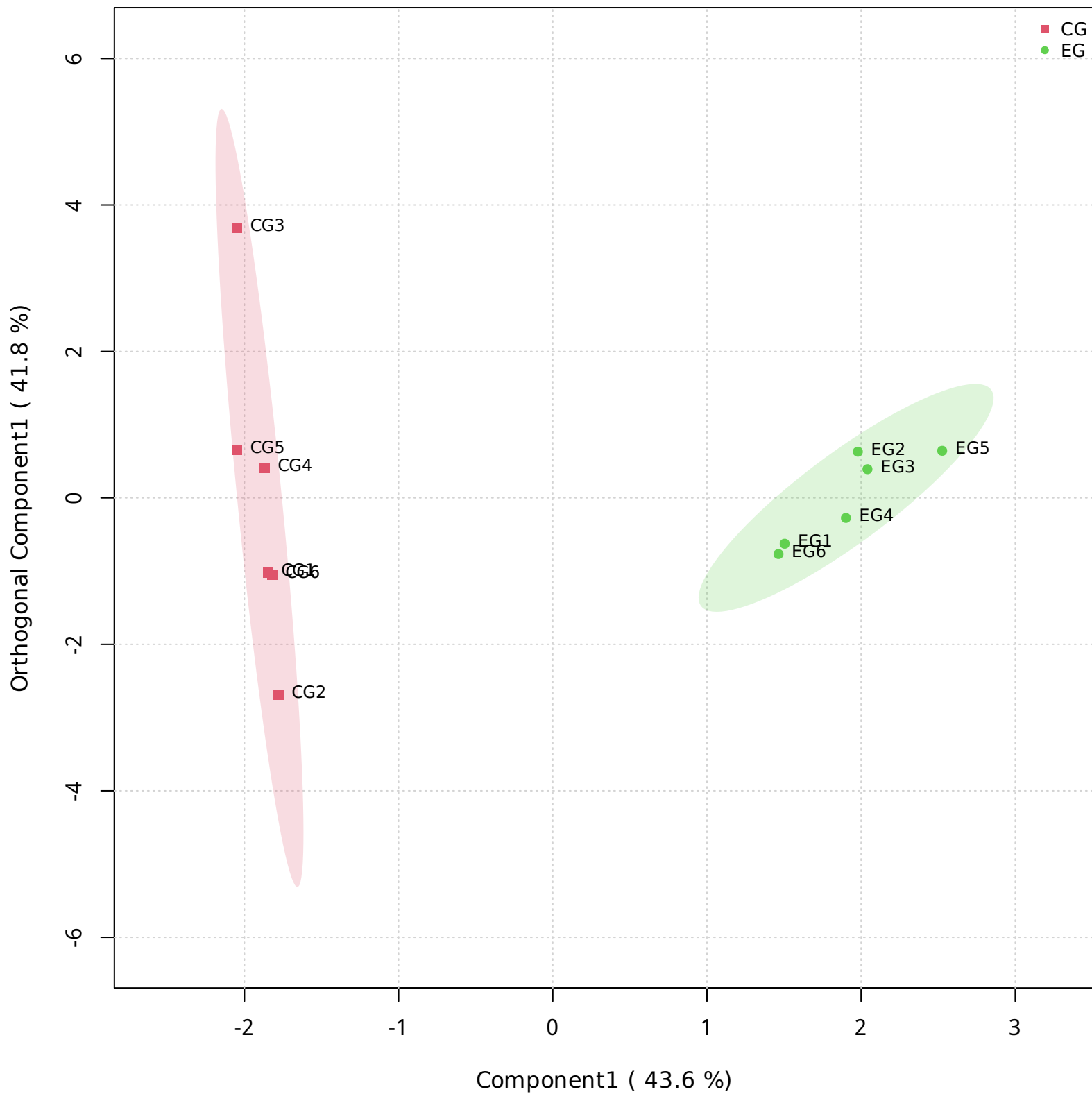

Supplement: Supplementary file 1 [file animals-16-01825-s001.zip › Figure S2.pdf]

# PCA Scores Plot

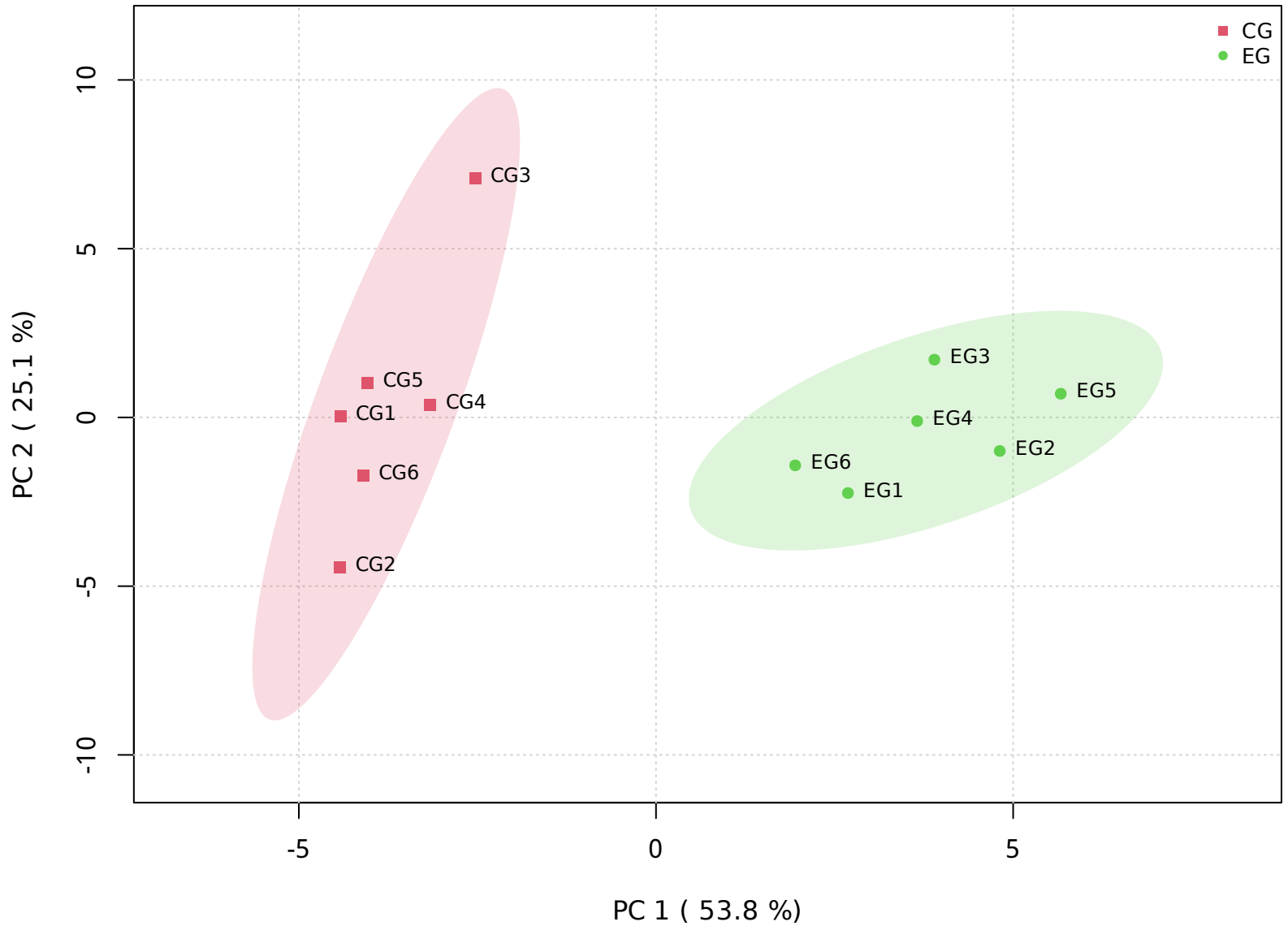

Supplement: Supplementary file 1 [file animals-16-01825-s001.zip › Figure S1.pdf]
